# Tideglusib improves novel object recognition memory in the preclinical DBA/2J *mdx* mouse model of Duchenne muscular dystrophy

**DOI:** 10.3389/fnins.2026.1812975

**Published:** 2026-04-22

**Authors:** Emily N. Copeland, Amélie A. T. Marais, Ahmad Mohammad, Bianca M. Marcella, Ryan W. Baranowski, Shawn M. Beaudette, Rebecca E. K. MacPherson, Val A. Fajardo

**Affiliations:** 1 Department of Kinesiology, Brock University, St. Catharines, ON, Canada; 2 Department of Health Sciences, Brock University, St. Catharines, ON, Canada; 3 Centre for Neurosciences, Brock University, St. Catharines, ON, Canada; 4 Centre for Bone and Muscle Health, Brock University, St. Catharines, ON, Canada

**Keywords:** Alzheimer’s disease, DMD, GSK3, recognition memory, tideglusib

## Abstract

**Introduction:**

Duchenne muscular dystrophy (DMD) is a severe X-linked neuromuscular disorder characterized by progressive muscle wasting. Approximately 1 in 3 DMD patients experience cognitive dysfunction, with research suggesting an Alzheimer’s disease (AD)-like pathology. We have previously shown that treatment with the glycogen synthase kinase 3β (GSK3) inhibitor, tideglusib, improves muscle quality, function, and insulin sensitivity in the DBA/2J (D2) *mdx* mouse model of DMD. In this brief follow-up study, we report the effects of tideglusib treatment on cognitive function.

**Methods:**

Male D2 WT and *mdx* mice were purchased from Jackson Laboratories. Mice were separated into the following groups: (1) WT, (2) *mdx*-vehicle, and (3) *mdx*-tideglusib (10 mg/kg/day via oral gavage for 4 weeks). A novel object recognition test was performed to assess recognition memory. Hippocampus and serum samples were collected for BACE1 activity assays, amyloid beta (Aβ) ELISAs, and western blotting.

**Results:**

Compared to vehicle-treated *mdx* mice, tideglusib-treated *mdx* mice demonstrated improved recognition memory. These changes to recognition memory were accompanied by greater expression of beta-catenin, an indirect downstream marker of GSK3 inhibition. While there were no changes in BACE1 activity, tideglusib-treated *mdx* mice had higher concentrations of Aβ in the serum and lower protein levels of receptor of advanced glycation end products.

**Discussion:**

The results from this brief follow-up study offer preliminary support for tideglusib as a treatment for both muscle and brain impairments in *mdx* mice, potentially improving cognitive function through enhanced vascular Aβ clearance.

## Introduction

Duchenne muscular dystrophy (DMD) is a severe X-linked muscle-wasting disorder resulting from the absence of functional dystrophin and affects approximately 1 in 5000 males worldwide (Mah et al., 2014). A substantial proportion of boys living with DMD (30%–40%) also present with cognitive impairments and neurobehavioural changes such as lower intelligence quotient scores (<70) (Cotton et al., 2001) and deficits in reading and learning (Dorman et al., 1988). Combined, impaired muscle and cognitive function can lower one’s quality of life. Therefore, reducing both muscle and brain pathology can drastically enhance the lives of those suffering from DMD.

Tideglusib is a non-ATP competitive glycogen synthase kinase 3 beta (GSK3β) specific inhibitor that has shown neuroprotective effects in both murine and cellular models (Fuchs et al., 2018; Armagan et al., 2017). Specifically, 20 days of subcutaneous injection of tideglusib has shown improved hippocampal development and dependent behaviour in young CDKL5 KO mice (Fuchs et al., 2018), and 50 days of intraperitoneal injection of tideglusib has shown protective effects against hypoxic ischemia in neonatal mice (Wang et al., 2016), highlighting its beneficial action on the central nervous system. Further, tideglusib has underwent clinical trials for myotonic dystrophy and Alzheimer’s disease (AD) (Lovestone et al., 2015; Horrigan et al., 2020), and we have previously shown that tideglusib improves skeletal muscle function in the *mdx* mouse (Marcella et al., 2024), making it a potentially viable whole-body therapeutic option.

In AD, the amyloid precursor protein (APP) is competitively cleaved by beta- and alpha-secretase (BACE1 and ADAM10, respectively). Cleavage of APP by BACE1 is the rate-limiting step in amyloid beta (Aβ) production, and previous work has shown that GSK3β overactivity in the brain increases BACE1-mediated cleavage of APP (Ly et al., 2013). We have previously shown that tideglusib treatment in DBA/2J (D2) *mdx* mice improved muscle health, strength, and endurance (Marcella et al., 2024). However, the effects of these treatments on cognitive function in these *mdx* mice remain unknown. This is important, as we and others have previously shown that *mdx* mice display cognitive dysfunction, with impaired recognition memory in a novel object recognition test (NORT), and exhibit an AD-like pathology (Bagdatlioglu et al., 2020; Hayward et al., 2022). Here, in this preliminary and exploratory follow-up study, we examined the effects of tideglusib treatment on recognition memory and markers of Aβ production in D2 *mdx* mice. We hypothesized that tideglusib treatment would improve recognition memory in D2 *mdx* mice and reduce AD-like pathological markers, including lower BACE1 activity and Aβ production in the hippocampus.

## Methods

### Animals

Male D2 *mdx* and wildtype (WT) mice were ordered from Jackson Laboratories (Bar Harbor, Maine, USA) at 7–9 weeks of age and were separated into three groups (*n* = 10 per group): (1) WT control, and *mdx* mice were randomized into: (2) *mdx* vehicle (*mdx*-vehicle), and (3) *mdx* tideglusib (*mdx*-tideglusib). Beginning at 24–26 weeks of age, the *mdx-*tideglusib group was treated with tideglusib at 10 mg/kg/day, consistent with the clinical dose of 600 mg for a 60 kg patient (Horrigan et al., 2020), and the *mdx-*vehicle group was treated with 20% Kolliphor (v/v) via oral gavage for 4 weeks. All mice were housed in Brock University’s Animal Facility in an environmentally controlled room on a standard 12:12 h light–dark cycle. Mice were allowed access to food and water *ad libitum*. All experimental procedures were approved by Brock University’s Animal Care Committee (AUP No. 22-08-03) and carried out in accordance with the Canadian Council on Animal Care guidelines.

### Novel object recognition test

The NORT was performed as previously described (Hayward et al., 2022; Baranowski, 2022; Townsend et al., 2025; Maheu et al., 2025). Briefly, all mice underwent a habituation period in which they were placed in a four-sided arena (40 × 40 × 40 cm) and allowed to explore freely for 10 min. Following the habituation period, mice were placed in a four-sided arena with two identical objects in opposing corners. Mice were allowed to explore for 10 min before being returned to their home cage for a four-hour intertrial retention delay period. Following the delay, mice were returned to the arena where one object was replaced with a novel object. Mice were allowed to explore for 10 min. All objects were cleaned between trials and were previously tested for innate preference and discrimination. Objects were counterbalanced across animals and trials. All trials were recorded using a ceiling-mounted GoPro (1920 × 1,080 pixels, 30 fps). Animal key point detection was performed using DeepLabCut (DLC version, 2.2). A custom DLC model was trained using user inputs on the locations of the four key points: nose, tail base, left ear, and right ear. The DLC model (ResNet 50 architecture) was trained using 20 diverse frames (kmeans clustering) from 20 input videos, resulting in 20, 000 epochs and resulting in a mean testing error of 3.74 pixels across the tracked landmarks. A custom MatLab (v2022a, The MathWorks Inc.) was used to analyze the tracked XY positional data and generated discrete performance parameters. All trials were inputted by a blinded researcher and scored based on the amount of time each mouse spent interacting with the target object were computer generated, reducing the risk of bias. Distance travelled and time spent in the corners of the arena throughout the trial were also analyzed.

### Tissue collection

All mice were euthanized via exsanguination under general anesthesia (vaporized isoflurane). The hippocampus and serum were collected and stored at −80 °C. Samples were homogenized at 1:10 weight/volume ratio in PMSF.

### BACE1 activity

BACE1 activity was determined using a fluorogenic enzymatic activity assay, as described (Mohammad et al., 2025). Samples were prepared at 0.5 μg/μL and 50 μL was added to a black 96-well plate in duplicate with 50 μL of assay buffer and 2 μL of beta-secretase substrate. The plate was incubated in the dark at 37 °C for 60 min before endpoint fluorescence was read using a spectrometer (SpectraMax, M2; Molecular Devices) at excitation and emission wavelengths of 350 and 490 nm, respectively.

### Aβ ELISA

Aβ concentration was determined using a commercially available kit (ThermoFisher, KMB3441) via sandwich ELISA. Hippocampal and serum samples were prepared at 0.5 μg/μL and 5 μg/μl, respectively, and plated in duplicate as directed on the provided plate. Absorbance was read at 450 nm using a spectrometer (SpectraMax, M2; Molecular Devices).

### Western blotting

Western blotting was performed on hippocampal homogenates to determine protein expression of total GSK3β (Cell Signaling, 9,315), phosphorylated GSK3β (Cell Signaling, 5,558), beta-catenin (Cell Signaling, 9,562), BACE1 (Cell Signaling, 5606P), ADAM10 (Abcam, ab1997), lipoprotein receptor-related protein-1 (LRP-1; Cell Signaling, 263,875), and receptor for advanced glycation end-products (RAGE; Cell Signaling, 425,445). Standard SDS-PAGE was performed using 7–13% TGX gels (BioRad, 4,561,086) and transferred to either polyvinylidene difluoride (PVDF; BioRad) or nitrocellulose membranes (BioRad). All proteins were blocked with Everyblot buffer (BioRad, 12,010,020) for 10 min at 25 °C and were incubated with primary (overnight at 4 °C; Supplementary Table S1) and secondary antibodies (1 h at 25 °C). Imaging was performed using BioRad ChemiDoc Imager with Immobilon ECL Ultra Western HRP substrate (Sigma-Aldrich, WBKLS0500) or SuperSignal West Femto Maximum Sensitivity Substrate (ThermoFisher, 34,096). All images were analyzed using BioRad ImageLab software and were normalized to total protein using a ponceau stain.

### Statistical analysis

All analyses were performed using GraphPad Prism 9 (San Diego, California, USA). Statistical analysis was performed using a one-way ANOVA and Tukey’s post-hoc test. Statistical outliers were identified using the ROUT method within GraphPad Prism, with *Q* = 5% across all experiments. Significance was set to *p* < 0.05. Eta squared (*η*^2^; small = 0.01, medium = 0.06, large = 0.14) was calculated to determine effect size (Richardson, 2011) and Cohen’s *d* (small = 0.20, medium = 0.50, large = 0.80) was calculated to determine the effect size of significant post-hoc analyses (Cohen, 1988). All values are represented as means ± SEM.

## Results

### Tideglusib treatment improves recognition memory

**Figure 1:**
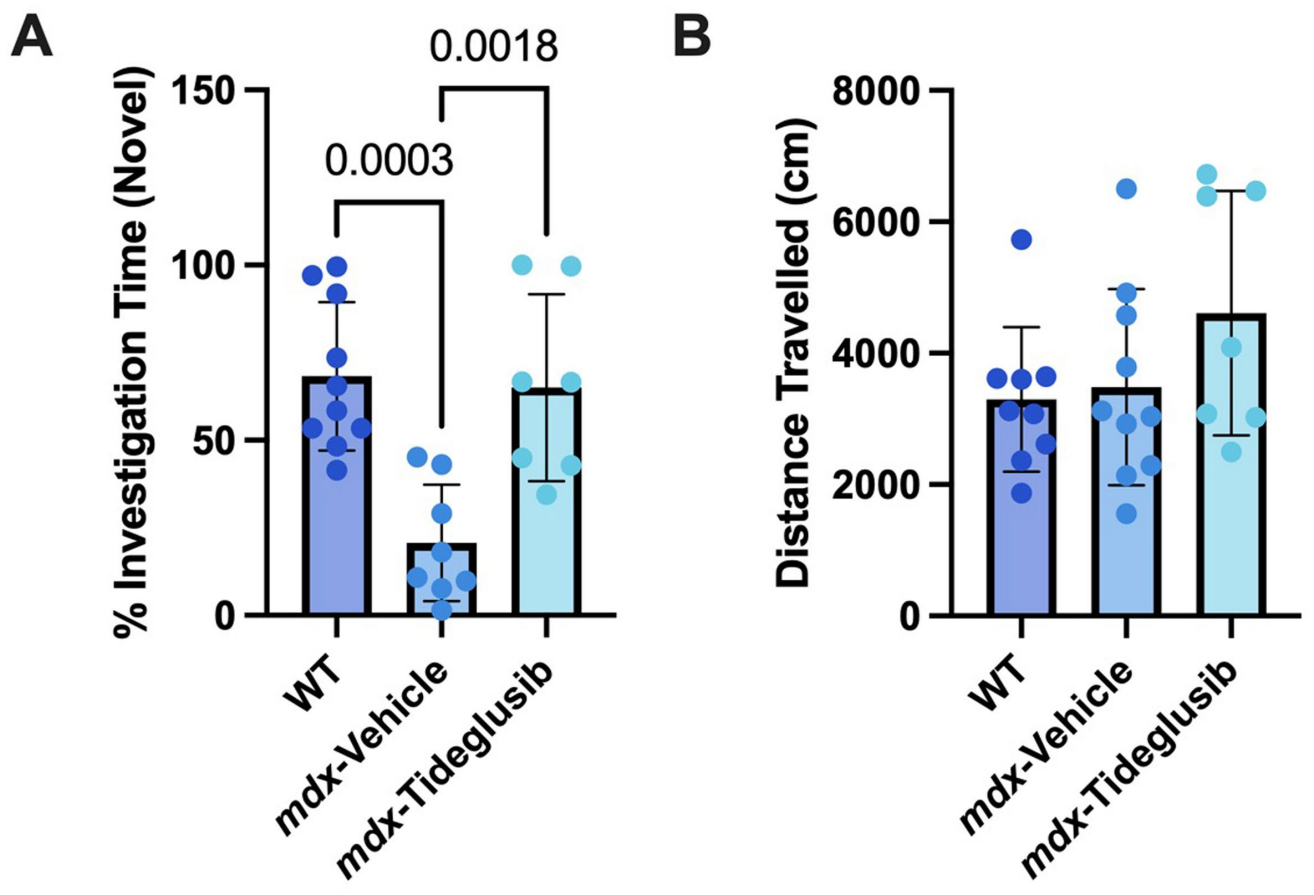
Tideglusib treatment improves novel object recognition memory in *mdx* mice. **(A)** Percent of investigation time spent with the novel object. **(B)** Distance covered throughout the investigation period in centimeters. All data represented as means ± SEM. A one-way ANOVA was run, and exact *p*-values are shown for statistically significant results. *n* = 6–10 per group.

We found that WT mice had better recognition memory compared with *mdx*-vehicle mice, spending more than double the amount of time investigating the novel object (Figure 1A; *p* = 0.0018, Cohen’s *d* = 2.4635). Moreover, the *mdx*-tideglusib mice showed improvements in recognition memory to levels similar to WT mice and significantly greater than *mdx*-vehicle mice (Figure 1A; *p* = 0.0003, Cohen’s *d* = 2.0327). These differences were not due to any differences in total distance covered throughout the investigation period (Figure 1B; *p* = 0.1945, *η*^2^ = 0.1327).

### Tideglusib does not change GSK3β phosphorylation status or protein expression

**Figure 2:**
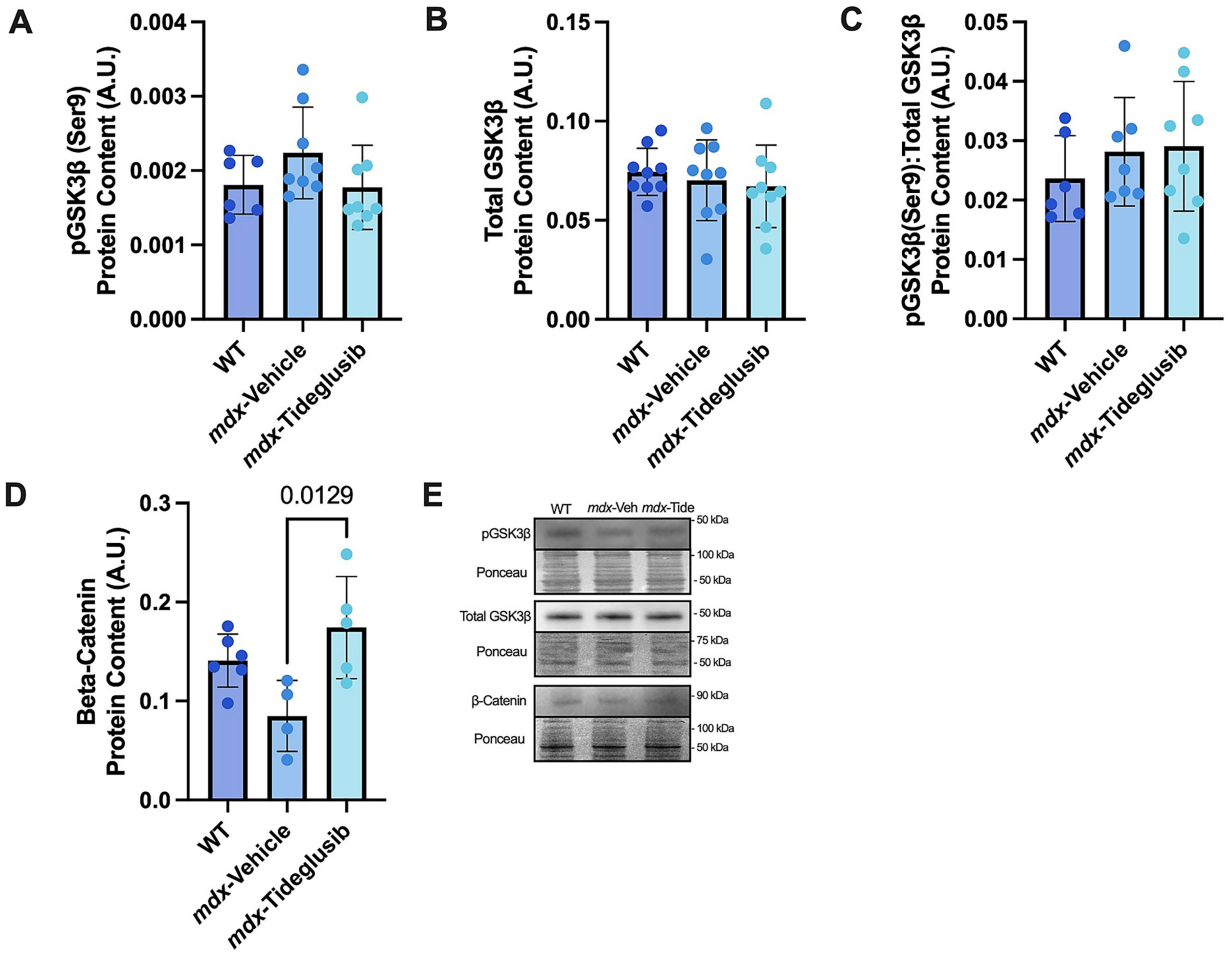
Tideglusib treatment does not change GSK3β phosphorylation status in hippocampus of *mdx* mice. **(A)** Western blot analysis of serine 9 phosphorylation of GSK3β (48 kDa, approximate molecular weight). **(B)** Western blot analysis of total GSK3β (48 kDa, approximate molecular weight). **(C)** Ratio of phosphorylated (serine 9) to total GSK3β protein expression. **(D)** Western blot analysis of beta-catenin (90 kDa, approximate molecular weight). **(E)** Representative images. All data represented as means ± SEM. A one-way ANOVA was run, and exact *p*-values are shown for statistically significant results. *n* = 68 per group.

The hippocampus is well known for its role in learning and memory and is heavily affected by AD-related pathologies. To examine GSK3*β* activity in the hippocampus, we measured total content and inhibitory phosphorylation (serine9). We did not observe any differences in total (*p* = 0.2699, *η*^2^ = 0.0294), serine9 phosphorylation (*p* = 0.5977, *η*^2^ = 0.1539), or the ratio between total and phosphorylated GSK3β (Figures 2A–C and E; *p* = 0.8723, *η*^2^ = 0.0651). Notably, tideglusib’s primary mode of inhibition is to irreversibly bind to GSK3β, holding it in an inactive state. Protein expression of beta-catenin, a downstream marker of GSK3β inactivity, was found to be significantly higher in the *mdx*-tideglusib mice compared to *mdx*-vehicle mice (Figures 2D and E; *p* = 0.0129, Cohen’s *d* = 0.3453). Collectively, these results suggest that tideglusib treatment did not alter total or phosphorylated GSK3β, yet still led to greater levels of β-catenin, which may potentially be an indirect marker of lower GSK3β activity.

### Tideglusib does not change AD-like pathogenic markers in the hippocampus

**Figure 3:**
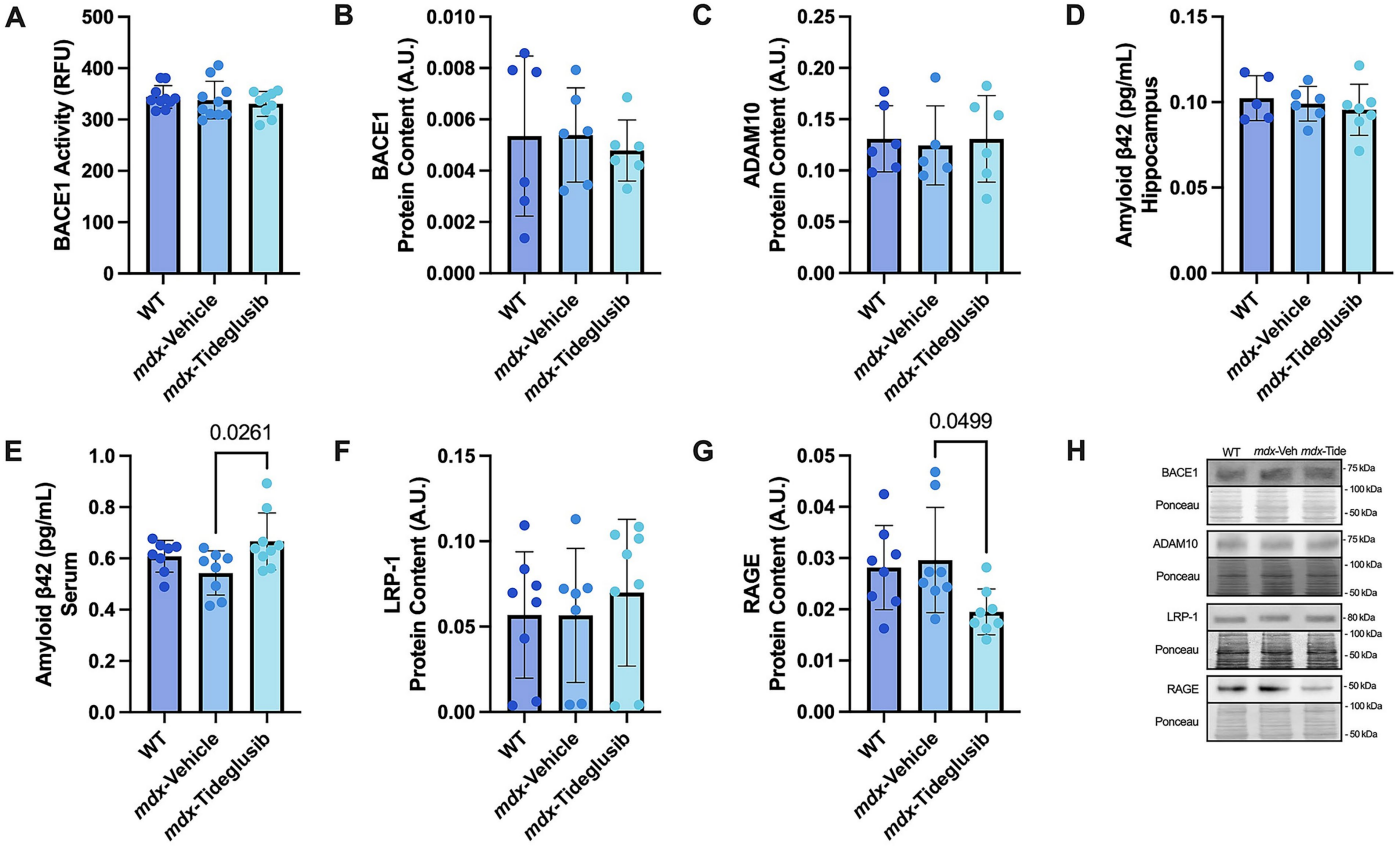
Tideglusib treatment does not change APP metabolic markers in hippocampus of *mdx* mice but may enhance Aβ handling. **(A)** BACE1 enzymatic activity. **(B)** Western blot analysis of BACE1 (75 kDa, approximate molecular weight). **(C)** Western blot analysis of ADAM10 (75 kDa, approximate molecular weight). **(D)** Aβ concentration in hippocampus. **(E)** Aβ concentration in serum. **(F)** Western blot analysis of LRP-1 (80 kDa, approximate molecular weight). **(G)** Western blot analysis of RAGE (50 kDa, approximate molecular weight). **(H)** Representative images. All data represented as means ± SEM. A one-way ANOVA was run, and exact *p*-values are shown for statistically significant results. *n* = 6–8 per group.

To investigate AD-like pathology, we examined the pathogenic marker BACE1, however, we observed no differences in its activity (*p* = 0.5943, *η*^2^ = 0.0392) nor protein expression between groups (Figures 3A,B and H; *p* = 0.8694, *η*^2^ = 0.0184). ADAM10 competitively cleaves APP in opposition to BACE1; nevertheless, its protein expression was also unchanged (Figure 3C and H; *p* = 0.9511, *η*^2^ = 0.0071). Consistent with the lack of alterations in these key regulators of Aβ production, we detected no differences in hippocampal Aβ levels between groups (Figure 3D; *p* = 0.8360, *η*^2^ = 0.265).

Vascular clearance of Aβ is critical in limiting neuronal Aβ toxicity (Xiang et al., 2015); and *mdx*-tideglusib mice exhibited higher concentrations of Aβ in the serum compared to *mdx*-vehicle mice (Figure 3E; *p* = 0.0261, Cohen’s *d* = 1.2254), which may indicate enhanced vascular clearance. Two proteins involved in regulating Aβ efflux and influx across the blood–brain barrier are lipoprotein receptor–related protein 1 (LRP-1) and the receptor for advanced glycation end-products (RAGE), respectively. While LRP-1 protein content was unchanged between groups (Figures 3F and H; *p* = 0.2869, *η*^2^ = 0.0278), RAGE protein levels were lower in *mdx*-tideglusib mice relative to *mdx*-vehicle (Figure 3G and H; *p* = 0.0499, Cohen’s *d* = 1.2774).

## Discussion

The purpose of this study was to provide a preliminary analysis of the effects of tideglusib on cognitive function in D2 *mdx* mice. Our results demonstrate that *mdx*-vehicle mice exhibited impaired recognition memory compared with WT mice, whereas *mdx*-tideglusib mice showed restored recognition memory, comparable to WT controls. This improvement was accompanied by increased hippocampal *β*-catenin levels, which may be indicative of effective GSK3β inhibition with tideglusib treatment, although β-catenin remains an indirect downstream marker. Notably, tideglusib did not alter N-terminal serine9 phosphorylation of GSK3β. However, serine9 phosphorylation is not a primary regulatory mechanism of GSK3β within the canonical Wnt signalling pathway and does not directly govern β-catenin accumulation (Li et al., 2023). Instead, GSK3β regulates β-catenin within the destruction complex, where its activity depends on its localization and interaction with β-catenin (Doble and Woodgett, 2003). Therefore, inhibitors such as tideglusib that directly bind to GSK3β, independent of serine9-mediated regulation, may still influence β-catenin levels.

Contrary to our hypothesis, GSK3β inhibition did not alter hippocampal Aβ levels or the activity or expression of key regulators of Aβ production, BACE1 and ADAM10. Instead, tideglusib treatment resulted in higher serum Aβ levels in *mdx* mice than in vehicle-treated *mdx* controls. When considered alongside the reduction in RAGE content—which is known to regulate circulating Aβ by facilitating its influx into the brain—these findings suggest that tideglusib may enhance Aβ handling in *mdx* mice, thereby contributing to improved cognitive function. However, this remains speculative and dedicated studies specifically examining vascular Aβ handling are required to confirm this proposed mechanism, particularly given the absence of changes in hippocampal Aβ content. Moreover, RAGE contributes to other aspects of AD pathology, including the formation of neurofibrillary tangles and impaired synaptic transmission (Cai et al., 2016), and whether these pathways are altered with tideglusib treatment requires further investigation.

Though preliminary, this study comes with limitations. We were limited in that only one cognitive test was performed. We have previously shown that *mdx* mice exhibit recognition memory impairments using the NORT (Hayward et al., 2022), and being that this was an exploratory study, we opted to use the same approach. However, given that there was improvement to recognition memory with tideglusib treatment, future studies should explore a wider battery of cognitive tests to broaden these findings. Though sufficient to improve dystrophic muscle pathology (Marcella et al., 2024) this is a preliminary study secondary to muscle-centric effects and therefore may be underpowered to detect all cognitive effects. Additionally, 4-week tideglusib treatment may be too short to elicit mechanistic changes in the hippocampus. In fact, although 6 weeks of tideglusib treatment in mild-to-moderate AD patients showed therapeutic efficacy, including positive trends on cognitive tests (del Ser et al., 2013), subsequent clinical trials showed improvements in mild AD patients only after 26-week treatment, with little clinical benefit as mechanistic markers were widely unchanged (Lovestone et al., 2015). In this regard, results from this study align well with clinical trials, however, longer dosing periods should be explored in the future to gain mechanistic insight to improvements in recognition memory.

We have demonstrated that tideglusib improves muscle strength, morphology, and metabolic health in *mdx* mice (Marcella et al., 2024), and recent work has highlighted the potential for a muscle to brain connection that might underlie cognitive benefits (Copeland et al., 2025; Brisendine and Drake, 2023; Brisendine and Drake, 2025). Therefore, it is possible that peripheral to central pathways may be contributing to the enhanced recognition memory observed in this study, although further work is needed to assess this hypothesis.

Although this study does not provide definitive mechanistic insight, it offers additional support for the therapeutic potential of tideglusib in DMD. In addition to improvements in muscle and metabolic health in D2 *mdx* mice (Marcella et al., 2024), with further investigation, our findings might suggest that tideglusib may also confer cognitive benefits. If translated clinically, simultaneous improvements in both muscle and brain health would substantially enhance quality of life for individuals living with DMD.

## Data Availability

The raw data supporting the conclusions of this article will be made available by the authors, without undue reservation.
