# Supplementary material for: Tideglusib improves novel object recognition memory in the preclinical DBA/2J mdx mouse model of Duchenne muscular dystrophy
Source: Front Neurosci. 2026 Apr 22;20:1812975. doi: 10.3389/fnins.2026.1812975 (PMC13144132; doi:10.3389/fnins.2026.1812975)
Supplement: Supplementary file 1 [file Table_1.DOCX]

**SUPPLEMENTARY MATERIALS**

**Supplemental Table 1.** Primary antibody concentrations.

| Primary Antibody | Concentration |
| --- | --- |
| GSK3β | 1:5000 |
| pGSK3β(Ser9) | 1:1000 |
| Beta-catenin | 1:2000 |
| BACE1 | 1:2000 |
| ADAM10 | 1:2000 |
| LRP-1 | 1:2000 |
| RAGE | 1:2000 |
